# The psychological processes of classic psychedelics in the treatment of depression: a systematic review protocol

**DOI:** 10.1186/s13643-022-01930-7

**Published:** 2022-05-05

**Authors:** Lauren Johansen, Paul Liknaitzky, Maja Nedeljkovic, Lisa Mastin-Purcell, Greg Murray

**Affiliations:** 1 Centre for Mental Health, Department of Psychological Sciences, Swinburne University of Technology, Melbourne, Australia; 2 Turner Institute (School of Psychological Sciences), Department of Psychiatry (School of Clinical Sciences), Monash University, Melbourne, Australia

**Keywords:** Psychedelic-assisted psychotherapy, Psilocybin, LSD, Depression, Psychological processes, Systematic review, Narrative synthesis

## Abstract

**Background:**

There is currently renewed interest in the use of psychedelic therapy in the treatment of psychiatric disorders, including depression. The proposed systematic review will aim to identify, evaluate and summarise the psychological processes of change underlying psychedelic therapy for depression in the current literature and consider the implications these processes may have on the psychotherapy component of treatment.

**Methods:**

Scopus, PsycINFO, PubMed and Web of Science databases will be searched using relevant terms. Studies will be included if they discuss the use of a classic psychedelic to treat depression symptomology in an adult population and report or propose psychological processes responsible for depression symptom change. Two authors will independently screen articles, complete quality assessment tools and conduct data extraction. Empirical and non-empirical research will be extracted and synthesised separately. A narrative synthesis approach will be used to report psychological processes identified in the literature.

**Discussion:**

This systematic review will be the first to collate available evidence on the psychological processes associated with psychedelic therapy for depression. The preliminary nature of this research field is expected to result in the review having several limitations, namely heterogeneity between studies and the inclusion of limited empirical research. We intend for this review to present the current state of the literature, identify gaps and generate candidate variables that warrant further investigation.

**Systematic review:**

PROSPERO CRD42020197202

## Background

Classic psychedelics are a group of psychoactive drugs that includes lysergic acid diethylamide (LSD), psilocybin, *N,N*-dimethyltryptamine (DMT) and mescaline [1]. When ingested or administered, these compounds can elicit altered states of perception, cognition and emotion [2]. Despite having distinct characteristics, the classic psychedelic compounds are often grouped together as they all act as agonists of serotonin 5-hydroxytryptamine (5-HT) 2A receptors [3]. It is through this receptor agonism that classic psychedelics primarily exert their psychoactive effects [1].

The use of classic psychedelics by non-Western cultures can be traced back thousands of years. The sacramental consumption of mescaline, via ingestion of the Peyote cactus (*Lophophora williamsii),* occurred in Native American ceremonies as far back as 5 millennia [4], and there is evidence to suggest that DMT-containing plants have been ingested by some in South America since 2130 BC [5]. Similarly, psilocybin-containing mushrooms may have been used in Mesoamerican ceremonies for at least 3500 years [6].

The synthesis and ingestion of LSD in 1942 [7] sparked Western scientific interest into psychedelics and led to numerous studies investigating the efficacy of LSD and psilocybin as a treatment for psychiatric disorders [2]. However, increasing restrictions to the access of psychedelics in the late 1960s, and the subsequent criminalisation of psychedelics in 1970, led to a significant decrease in human psychedelic research in the following 20 years [8, 9]. It was not until the 1990s that ethics approval was granted for several healthy participant studies that utilised psychedelics with less notoriety than LSD, including DMT, psilocybin and mescaline [10–13]. This paved the way for further human trials to be conducted.

Recent clinical trials have reported promising results on the clinical benefits of classic psychedelics across a range of psychiatric disorders, including depression [14]. These trials have mostly studied psychedelic-assisted psychotherapy, whereby a psychedelic is administered in conjunction with psychological support or therapy. An open-label feasibility study reported significant symptom reductions in participants with treatment-resistant depression (TRD; defined as experiencing no improvements in depression symptoms despite two courses of antidepressant treatment) 1 week post-treatment [15]. Symptom reductions remained significant 6 months following treatment [16]. These findings have been further supported by randomised trials, which found psilocybin-assisted psychotherapy to produce significant reductions in depression symptomology in individuals with major depressive disorder (MD D[17, 18];) and those experiencing distress associated with life-threatening cancer [19, 20].

Similar investigations have been conducted with ayahuasca, a brew containing DMT from the *Psychotria viridis* plant and monoamine oxidase inhibitors from the *Banisteriopsis caapi* vine. These studies have included participants currently experiencing a mild to severe depressive episode and had inadequately responded to at least one courses of antidepressants [21–23], as well as those experiencing depression and anxiety whilst attending a residential drug treatment centre [24]. These open label [22–24] and randomised control [21] trials have suggested that ayahuasca may produce rapid and significant reductions in depression symptoms in as little as 1 day.

The symptom changes engendered by psychedelic-assisted psychotherapy appear to occur rapidly following treatment, unlike the weeks or months typically seen in current first line treatments, such as antidepressant medication or psychotherapy [25]. A recent randomised controlled trial found psilocybin-assisted psychotherapy and escitalopram to have similar anti-depressant effects for individuals with MDD [17]. Whilst no significant difference was found in the primary depression outcome between the two treatments, psilocybin-assisted psychotherapy appeared to display superior tolerability and greater improvements in secondary depression outcomes, in comparison to escitalopram [17]. Participants who received psilocybin-assisted psychotherapy also reported greater improvements in wellbeing and anxiety scores [17]. In addition to rapid symptom reduction, preliminary findings from two long-term follow-ups have suggested that reductions in depression symptomology immediately following psilocybin-assisted psychotherapy are sustained 6 months post-treatment [16, 19].

Whilst psychedelic-assisted psychotherapy appears to be a promising treatment for depression, among other psychological disorders, there are several caveats that must be mentioned. Many of the trials completed to date have been open label or pilot studies, with small sample sizes. Other than the recent randomised controlled trial comparing psilocybin-assisted psychotherapy and escitalopram [17], no trial has utilised an active control. Studies which utilise a placebo or a non-psychoactive control face the challenge of participants being able to identify the substance they have consumed due to the psychoactive nature of psychedelics. Further studies utilising active controls in much larger samples are needed to support the current research into psychedelic-assisted psychotherapy for depression. Further longitudinal data is also required.

### Neurobiological processes in psychedelic treatment

Exactly how classic psychedelics may work to reduce depression symptoms is still largely unknown, although several potential mechanisms have been proposed. Increased functional connectivity within the default mode network (DMN), a group of brain regions which show increased activity during rest and decreased activity during cognitively demanding tasks, as well as decreased functional connectivity between executive networks and the DMN, are associated with the pathophysiology of MDD [26, 27]. A number of studies have demonstrated that classic psychedelics acutely reduce activity and internal functional connectivity within the DMN [28, 29], potentially reducing abstracted (perceptually decoupled) and self-referential rumination, a hallmark of depressotypic thinking [30–32]. However, similar changes in DMN connectivity have been observed post-administration of sertraline [33], a widely used selective serotonin reuptake inhibitor, suggesting that these changes may not be psychedelic-specific [34]. There is some evidence to suggest that the psychedelic state produces co-activation between the DMN and task positive networks (a network of brain regions which activate during attention demanding tasks), two networks which are usually anti-correlated [35]. However, these findings have not been replicated in a more recent investigation into the impact of ayahuasca on the DMN [28] so it is unclear as to whether this co-activation may be contributing to the novel experiences and outcomes which occur during psychedelic-assisted psychotherapy [35].

Several other neurobiological mechanisms of action have been suggested to contribute to the antidepressant effects of psychedelics. The amygdala has been found to show greater responsiveness to emotional faces 1 day after psilocybin consumption and greater responsiveness to fearful versus neutral faces was related to improved depressive symptoms 1 week post-intervention [36]. This may indicate that psilocybin has a direct effect on apathy symptoms associated with depression. Additionally, decreased functional connectivity between the amygdala and ventromedial prefrontal cortex have been found during face processing tasks 1 day post-psilocybin consumption [37]. During the same task, the amygdala and ventromedial prefrontal cortex were found to have increased functional connectivity with the occipital lobe and parietal cortex [37]. Taken together, these preliminary findings have been speculated to suggest that psilocybin may encourages increased emotional sensitivity and acceptance 1 day post-treatment [37].

Animal studies have suggested that psychedelics increase neuroplasticity, possibly through increased cortical glutamate, brain-derived neurotrophic factor, and activation of the tropomyosin receptor kinase B [34, 38, 39]. Other animal studies have proposed that psychedelics may elicit their antidepressant effect through anti-inflammatory processes, by inhibiting proinflammatory cytokines TNF-α and IL-6, which have been found to be associated with depression [40]. However, more research is required to comprehensively understand how psychedelics may be acting on the brain to alleviate depressive symptoms.

### Set and setting: psychological processes in psychedelic-assisted psychotherapy

It is important to note that the context in which classic psychedelics are used requires significantly greater consideration than traditional depression pharmacotherapies. This is due to both the positive therapeutic effects of the interpersonal context in which consumption takes place [41], as well as a number of risks associated with psychedelic consumption. These risks include transient anxiety, panic and, very rarely, prolonged psychosis [42]. It has long been recognised that both *set* (the mindset of the participant, including their expectations, assumptions and intentions for the psychedelic experience, as well as stable variables such as personality and the presence of psychopathology) and *setting* (the physical, social and cultural environment in which the experience occurs) have the potential to greatly impact the outcomes of the psychedelic experience [43, 44]. Psychedelic-assisted psychotherapy aims to account for this by conducting psychotherapy with participants to ensure they approach psychedelic therapy with a suitable set.

When undertaking psychedelic-assisted psychotherapy, participants take part in several sessions of psychotherapy before their psychedelic session/s. The purpose of these sessions includes preparing participants for what they may experience during a dosing session and constructive ways to respond to such experiences, establishing rapport between participant and therapists and setting beneficial expectations and intentions for the experience [42]. Additional sessions are provided following the psychedelic session/s, to assist in integrating their psychedelic experience once it is complete [45]. The therapeutic modality or style used has varied between trials. An acceptance and commitment therapeutic framework has been utilised in a trial with a depressed sample [17, 46]. Trials investigating the efficacy of psychedelic-assisted psychotherapy to assist smoking cessation and alcohol dependence have also employed cognitive behavioural therapy [47] and motivational enhancement therapy [48], but these modalities are yet to be utilised in a depression sample.

Contemporary clinical trials have also addressed setting by providing a comfortable and aesthetic environment, in addition to music and eye masks, for the psychedelic session to take place [42]. During these dosing sessions, a non-directive therapeutic style is adopted, with therapists instructing participants to ‘focus inward’ [49]. They may also provide support or encouragement for participants to engage with any challenging thoughts or memories that emerge [49]. This approach differs from the psycholytic therapy approach often utilised historically, whereby low to moderate doses of a chosen drug were supplied numerous times in the context of psychotherapeutic sessions [45]. Psycholytic therapy employed psychedelics to facilitate psychotherapeutic processes which may be challenging for the client to engage with, such as re-experiencing a past trauma [45]. Other historical models which utilised psychedelics includes the psychedelytic approach, which involves several psychotherapy sessions involving low doses of a psychedelic, followed by a session involving a large psychedelic dose [50]. These other models have yet to be utilised in modern trials and, as such, it is currently unclear as to how psycholytic or psychedelytic approach compares to the psychedelic-assisted psychotherapeutic model, in terms of efficacy, safety and practical applicability.

As indicated by the above procedures, the psychological and social context in which psychedelic-assisted psychotherapy occurs is of particular importance. This raises questions regarding the potential psychological processes which may be contributing to changes in depression symptoms. A focal point of this research area to date has been investigating mystical-type experiences, which includes the occurrence of “profound unity with all that exists, a felt sense of sacredness [and] a sense of the experience of truth and reality at a fundamental level” [13]. This experience is often operationalised into four factors: mysticism (a sense of unity, sacredness and noetic quality), positive mood, transcendence of time and space, and ineffability (difficulty in describing the experience [41];). Undergoing a mystical-type experience during the acute effects of a psychedelic has been associated with healthy volunteers reporting an improvement in their wellbeing [51, 52], and reductions in depression symptoms in participants with life-threatening cancer [19, 20].

The emotional breakthrough is a more recently conceived variable that may be a relevant process in psychedelic-assisted psychotherapy. It is described as the experience of facing challenging emotions and memories, and being able to ‘breakthrough’ these difficulties to find a sense of relief or resolution [53]. Naturalistic studies have suggested that greater experiences of an emotional breakthrough whilst under the influence of a psychedelic positively predict improvements in psychological wellbeing in non-clinical populations [53]. One study conducted with individuals reporting a lifetime diagnosis of an eating disorder provided some evidence that greater experiences of an emotional breakthrough related to greater reductions in depression symptomology following a psychedelic experience [54]. This finding was only at trend level and was not statistically significant. Indeed, substantially more research is required to understand how an emotional breakthrough effects the therapeutic outcomes of psychedelic-assisted psychotherapy, especially in depressed samples, but it appears to be a promising area for further exploration.

Several psychological processes occurring in the short-term following psychedelic consumption as well as during and after the psychotherapeutic component have also been identified. Through prospective surveys, Zeifman and colleagues [55] found experiential avoidance (the attempt to avoid or ignore negative or unwanted emotions or cognitions [56];) to be significantly reduced at 2 and 4 weeks after psychedelic use. Reductions in experiential avoidance were significantly associated with decreases in depression and suicidality scores [55]. These outcomes are in line with previous reports from participants of clinical trials, who described a notable shift from emotional avoidance to acceptance, after receiving psilocybin-assisted psychotherapy for treatment-resistant depression [57]. Other prospective surveys have found psychological flexibility (a set of processes which allow one to adapt to various demands, maintain balance across various domains and be open to and committed to behaviours congruent to their values) to be negatively correlated with depression scores post psychedelic use [58, 59]. Post-psychedelic decreases in neuroticism and increases in extroversion, and openness to experience have also been reported [60, 61]; however, these findings have been inconsistent across studies. Many of these processes have been studied through surveying individuals who ‘self-medicate’ with psychedelics, away from clinical trials. Further investigation into these processes, particularly in the context of clinical trials, is required.

Little is currently known about the importance of these, and other potential psychological mechanisms involved in psychedelic-assisted psychotherapy for depression. Given the importance of the psychological and social context in the delivery of this treatment, knowledge advancement in this area has the potential to strengthen treatment outcomes and minimise associated risks. A greater understanding of the underlying psychological processes may allow for treatment protocols to be refined, ensuring the most effective and safe treatment is delivered. One method of advancing our understanding is to conduct a thorough systematic review of the current literature, which allows the collatation evidence, uncovering of gaps in the literature, and facilitation of future research. Whilst current reviews, such as the review conducted by Mertens and Preller [62], offer comprehensive synthesises on neuropsychological mechanisms of action, there is yet to be a review focused exclusively on the psychological processes involved in the antidepressant effects of psychedelic-assisted psychotherapy. Therefore, the overarching aim of this systematic review will be to identify how classic psychedelics may operate at the psychological level to improve depression symptoms. The review will address two main questions: (i) what psychological processes of psychedelic therapy for depression symptoms can be discerned in existing literature, and (ii) what implications do proposed psychological processes have for the psychotherapy component of psychedelic-assisted psychotherapy?

## Method

The systematic review has been registered with the International Prospective Register of Systematic Reviews (PROSPERO, http://www.crd.york.ac.uk/PROSPERO, registration number: CRD42020197202). It will follow the Preferred Reporting Items for Systematic Reviews and Meta-Analyses Protocol (PRISMA-P) recommendations for systematic review protocols [63] and findings will be reported in accordance to PRISMA guidelines [64].

### Criteria for study inclusion

#### Study methods

All study methods, including experimental designs, reviews, theoretical and conceptual analyses, will be sought. It is recognised that broadening the inclusion criteria to allow all levels of knowledge may reduce the quality of evidence included in the review. However, as this is an emerging field, it is anticipated that there will be few randomised controlled trials or open label studies. We aim to offset these concerns by conducting separate data extraction forms and synthesis for empirical and non-empirical data. Included articles must be published in a peer-reviewed journal. Studies will be excluded if they are written in a language other than English, were published before 1990 or are conducted with animals. Earlier research will be excluded due to anticipated challenges in synthesising and comparing data from this era, due to vastly different conceptualisations of depressive symptoms and language used to describe psychological processes.

##### Population

To be eligible for inclusion, empirical studies must collect data measuring depressive symptoms in adults. We will not require studies to include participants with clinically elevated symptoms or a diagnosis of a depressive disorder. A variety of symptom measurement approaches will be accepted (e.g. self-report, standardised measures, interviews) and the psychometrics of the approach will be considered when reporting outcomes. Studies which include participants with co-occurring mental disorders or symptoms will be included. Data extraction and synthesis will occur separately for studies where depression symptomology is the primary focus of the research and those which consider other disorders or symptoms to be their primary outcome.

##### Intervention

Studies must report on or discuss the use of a classic psychedelic (LSD, psilocybin, DMT/ayahuasca or mescaline) in treating depression symptoms. To focus the review, papers will be excluded if they only consider the use of psychedelics in the context of microdosing. Microdosing is the consumption of a ‘subthreshold’ dose of a psychedelic, so that minimal acute effects of the drug are experienced [65]. A microdose is usually defined as being between 1/10 and 1/20 of a ‘recreational’ psychedelic dose [65]. Based on this guide, studies will be excluded if they include a dose equal to or lower than 13 μg for LSD, 0.4 g for psilocybin, 6 μg for DMT, 15 ml of ayahuasca and 40 mg of mescaline [22, 66]. Whilst this review aims to uncover psychological mechanism of action, studies will be included even where the tested intervention did not include adjunctive psychotherapy or emotional support, as meaningful data/commentary on psychological mechanisms of psychedelics may be provided.

##### Outcomes

To be included in the review, studies must report on or propose psychological processes of psychedelic therapy in the treatment of depression. We expect that such variables may be identified from a range of sources in the literature, including statistical analysis, qualitative interviews or inferences from theoretical analysis.

#### Procedures

##### Search strategy

Scopus, PsycINFO, PubMed and Web of Science databases will be searched to identify potential studies. The search will be restricted to only include publications from 1990 to present. Following screening, a search for potentially suitable but uncaptured articles will be run through a backward search of the reference list of included articles, as well as a forward search of articles which have cited the selected articles. Database searches will be saved, and alerts will be used to notify authors of the publication of new articles which match the searches. An example of the search strategy to be used for Scopus can be found in Table 1.

**Table 1:** Example search strategy in Scopus

TITLE ( psychedelic OR hallucinogen OR tryptamine OR phenethylamine OR lsd OR "Lysgeric acid diethylamide" OR psilocybin OR psilocin OR psilocybe OR "magic mushrooms" OR dmt OR "N,N-DMT" OR “N,N-Dimethyltryptamine” OR dimethyltryptamine OR ayahuasca OR “Banisteriopsis caapi” OR “Banisteriopsis” OR “Psychotria viridis” OR hoasca OR mescaline OR peyote OR “San Pedro” OR ceremony* OR psychotomimetic OR psilocibin OR psilocybine OR “3,4,5-Trimethoxyphenethylamine” OR trimethoxyphenethylamine AND NOT microdos* AND NOT “dance movement therapy” AND NOT “disease modifying therapy	
AND ALL ( depress* OR "mood disorder" OR “affective disorder” OR “psychopathology” OR “mental illness” OR “mental health” OR “treatment resistant” OR “major depressive disorder” OR “depress* symptom” OR suicide*	
AND ALL ( "psych*proces*" OR mediator OR moderator OR mechanism OR "mechanism of change" OR “mechanism of action” OR affect OR avoidance OR acceptance OR mindful* OR "emotional process*" OR "therapeutic potential” OR “think*” OR “emotional state” OR decentering OR values OR “psych* flexibil*” or cognit* OR motivation OR personality OR “personality style” OR avoidance OR wellbeing OR “quality of life” OR “self-concept” OR “behavio* activat*” OR set OR setting OR rumination OR perception OR reinforc* OR spirit* OR open* OR “experiential breakthrough” OR “ego dissolution” OR “ego death” OR “mystical experience” OR “mystical-type experience” or “emotional breakthrough” OR “quality”)	

##### Study selection

Identified articles will be collated into Rayyan [67] and duplicates removed. Article selection will be completed by two reviewers (LJ and LMP) and take place in two steps. The first stage will involve titles and abstracts being screened against inclusion and exclusion criteria. Results from each reviewer’s study selection will then be compared and full texts will be reviewed if discrepancies occur. During the second stage, the full text of the remaining articles will be screened against inclusion and exclusion criteria. Cohen’s Kappa for inter-rater reliability will be assessed and reported. A third reviewer (GM) will make the final decision if agreement cannot be reached at either stage.

##### Data extraction

Data extraction will be completed independently by two reviewers (LJ and LMP) using Excel spreadsheets. Both reviewers will pilot these forms on five studies initially, with amendments being made after this if necessary. As substantial heterogeneity is expected between studies, included studies will be categorised into three distinct groups, each with an individual data extraction form. These groups will be: empirical research where depression symptomology is the primary target, empirical research where symptomology other than depression is the primary target, and non-empirical research. Data to be extracted from the empirical studies will include participant demographic information, sample size, type and severity of depression, method of measuring depression symptoms and psychological processes, other diagnosis/symptomology, study design and methodology, psychedelic used, the number of doses administered, the intervention type paired with the psychedelic, outcome data (e.g. changes in depression, changes in psychological processes) and psychological processes. The form for non-empirical work will focus on proposed psychological processes and the theory or evidence cited to support these variables.

Both reviewers will then conduct quality assessments on each article. A modified version of Murad and colleague’s methodological quality assessment tool will be used to assess quantitative studies [68]. This tool examines the quality of participant selection methods, ascertainment of exposure and outcomes, how causality was determined and the reporting of outcomes. Qualitative studies will be appraised using Dixon-Woods and colleague’s tool for appraising qualitative research [69]. This instrument examines the clarity of research questions, methodology and data integration, the suitability of the qualitative methodology used to answer the research questions, as well as the appropriateness of the conclusions drawn. If an agreement on the quality of an article cannot be reached, a third reviewer (GM) will be used.

##### Data synthesis

Due to heterogeneity, as well as the anticipated lack of empirical, quantitative research, a narrative synthesis will be used in conducting this review. A modified version of Popay and colleagues [70] framework for developing a narrative synthesis will be used. Synthesis will occur within the three distinct groups outlined in the data extraction section. In the first step, a preliminary synthesis of the outcomes from included studies will be reported. The primary aim of this stage will be to report the proposed psychological processes of psychedelic therapy, the direction of their effect and other relevant design features (e.g. participant demographics, the psychedelic administered) of each included article. Where multiple publications from the same study or dataset exist, these will be narrowed into a single summary. Next, a between study analysis will be undertaken. This will involve an exploration of how proposed processes may overlap with one another, followed by comparing the psychological processes reported by each study, and analysing how they may, or may not, be working together to change depression symptoms. In the final step, the robustness of the synthesis will be assessed. This will involve analysing the quality assessment results produced during the data extraction stage, study designs and the quality of measures used (e.g. method of measuring depression symptoms) to determine if some findings should be weighted more highly than others.

## Discussion

There are currently no systematic reviews exploring the psychological processes of classic psychedelic therapy in the treatment of depression symptomology. To date, reviews have focused on establishing treatment efficacy [30], understanding the neurological mechanisms of change [71], or have been broad in their aims (e.g. including a variety of drug classes or disorders [72, 73];). A review on the psychological processes is needed so current evidence can be collated and gaps in the literature identified. Furthermore, it is important to prompt further research into psychological processes alongside the existing focus on treatment efficacy. Research into other therapies for psychological disorders has heavily focused on establishing treatment efficacy [56]. While this is obviously important, the lack of focus on understanding how the intervention may be working has led to poorly understood treatments where the ‘active’ components are unknown. Placing greater importance on researching the underlying processes of treatments may allow for the development of interventions that are maximally efficient and effective [74]. The present review aims to address these shortcomings of previous psychological treatment research in the field of psychedelic therapy.

### Limitations

It is anticipated that the proposed systematic review will have several limitations. Heterogeneity between included studies, such as differences in study design, the psychedelic used, dosage and method of measuring depression symptoms, is expected. This may limit the capability of synthesising study outcomes in a meaningful way. Heterogeneity, as well as the anticipated lack of empirical, quantitative research, has meant a narrative synthesis will be used in conducting this review. Narrative synthesises are at increased risk of being biased by researcher’s opinions, which we intend to minimise through the rigorous methods detailed in this protocol. Finally, we acknowledge that, as this body of research is in its preliminary stages, this review will by no means provide a comprehensive and definitive understanding of the psychological processes occurring in psychedelic therapy for depression. Rather, we aim to present the current state of the literature and generate candidate variables that warrant further investigation.

## Abbreviations

LSD: Lysergic acid diethylamide
DMT: N, N-Dimethyltryptamine
DMN: Default mode network
MDD: Major depressive disorder
TRD: Treatment-resistant depression
